# Fragmentation and transferability in Hirshfeld atom refinement

**DOI:** 10.1107/S2052252522000690

**Published:** 2022-02-26

**Authors:** Michał Chodkiewicz, Sylwia Pawlędzio, Magdalena Woińska, Krzysztof Woźniak

**Affiliations:** a Biological and Chemical Research Centre, Department of Chemistry, University of Warsaw, Żwirki i Wigury 101, Warszawa 02-089, Poland

**Keywords:** Hirshfeld atom refinement, fragmentation, transferability, quantum crystallography

## Abstract

Hirshfeld atom refinement (HAR) is one of the most effective methods for obtaining accurate structural parameters for hydrogen atoms from X-ray diffraction data. Unfortunately, it is also relatively computationally expensive, especially for larger molecules due to wavefunction calculations. Here, a fragmentation approach has been tested as a remedy for this problem. It gives an order of magnitude improvement in computation time for larger organic systems and is a few times faster for metal–organic systems at the cost of only minor differences in the calculated structural parameters when compared with the original HAR calculations. Fragmentation was also applied to polymeric and disordered systems where it provides a natural solution to problems that arise when HAR is applied. The concept of fragmentation is closely related to the transferable aspherical atom model (TAAM) and allows insight into possible ways to improve TAAM. Hybrid approaches combining fragmentation with the transfer of atomic densities between chemically similar atoms have been tested. An efficient handling of intermolecular interactions was also introduced for calculations involving fragmentation. When applied in *fragHAR* (a fragmentation approach for polypeptides) as a replacement for the original approach, it allowed for more efficient calculations. All of the calculations were performed with a locally modified version of *Olex2* combined with a development version of *discamb2tsc* and *ORCA*. Care was taken to efficiently use the power of multicore processors by simple implementation of load-balancing, which was found to be very important for lowering computational time.

## Abstract 2

Hirshfeld atom refinement (HAR) combined with fragmentation and atomic density transferability was tested on various systems, both polymeric and disordered. HAR was significantly faster with these adjustments, reducing computational time for larger systems with only a modest drop in accuracy.

## 1. Introduction

Hirshfeld atom refinement (HAR, Hirshfield, 1977 ▸; Jayatilaka & Dittrich, 2008 ▸) is a leading method for accurate determination of hydrogen atom structural parameters from X-ray diffraction data which greatly outperforms traditional models based on spherical atomic densities (Capelli *et al.*, 2014 ▸; Woińska *et al.*, 2016 ▸; Malaspina *et al.*, 2017 ▸) called the independent atom model (IAM). Atomic form factors in HAR are based on wavefunctions for a given system and they include the effects of chemical bonding and intermolecular interactions. The strengths of these effects are most noticeable for hydrogen atoms. They are, however, neglected by the IAM and as a result the lengths of covalent bonds to hydrogen are on average shorter by *ca* 0.1 Å than the corresponding values derived from neutron diffraction experiments and hydrogen atom displacement refinement parameters usually lead to non-positive definite ADPs. Inclusion of the aspherical part of the atomic density in HAR leads to much better results – it is possible to reach a bond length and ADP precision comparable to that of neutron data (*e.g.* Capelli *et al.*, 2014 ▸). Although tailored quantum mechanical calculations are a source of the strength for the method, they are also relatively slow calculations, especially for large systems. Fortunately, quantum chemistry presents several solutions to the problem (Akimov & Prezhdo, 2015 ▸), including application of semiempirical methods (Christensen *et al.*, 2016 ▸), dividing systems into parts and applying mixed quantum-mechanical molecular calculations (Senn & Thiel, 2009 ▸). Alternatively, a method which seems to be the best suited for HAR can be applied: fragmentation (see *e.g.* Gordon *et al.*, 2012 ▸; Collins & Bettens, 2015 ▸; Raghavachari & Saha, 2015 ▸; Herbert, 2019 ▸), which involves dividing a larger system into smaller ones and performing calculations on those (fragments). Recently, this type of approach (referred to as fragHAR) was used by Bergmann *et al.* (2020 ▸) in HAR by applying a variant of fragmentation called molecular fractionation with conjugate caps (MFCC) introduced by Zhang & Zhang (2003 ▸). This approach was designed for protein systems, was tested on oligopeptide systems and showed good agreement in the resulting structural parameters with HAR. In fact, the method of electron density calculation proposed within the original MFCC scheme (Gao *et al.*, 2004 ▸) differs from that used in fragHAR, although they both divide the polypeptide into overlapping fragments in the same way. Quantum chemical calculations for elastic X-ray scattering with fragmentation were also recently applied by Northey & Kirrander (2019 ▸). Most fragmentation approaches focus on obtaining accurate energies, which are global properties, but in the case of HAR, the calculations provide electron density (a local property), making the fragmentation scheme for HAR less complicated. The main goal of fragmentation is to accelerate the quantum mechanical calculations and make them quite easy to achieve for very large systems. This does not necessarily have to be the case for small- to medium-sized molecules, especially when parallel computing is used. Indeed, in the case of fragHAR, the maximum time saved was quite modest as fragHAR calculations were about 2× faster than regular HAR for the largest tested system, a hexapeptide (C_22_H_36_N_6_O_7_), and even less (∼1.5 times) in the case of parallel execution.

Although HAR seems to be the most accurate approach for obtaining the structural parameters of hydrogen atoms from X-ray diffraction, there are also other – faster than HAR – approaches reaching beyond the IAM and significantly outperforming it in terms of structural parameter accuracy. One of the most well established approaches is the transferable aspherical atom model (TAAM) based on the Hansen–Coppens multipole model (Hansen & Coppens, 1978 ▸) and takes advantage of the fact that the parameters of this model are similar for atoms in similar chemical environments (Pichon-Pesme *et al.*, 1995 ▸) and therefore they can be stored in databanks and (re)used in refinements. The model allows for free-refinement of hydrogen positions and produces much more accurate structural parameters than IAM, as shown previously for a number of databanks of multipole model parameters (Zarychta *et al.*, 2007 ▸; Domagała *et al.*, 2012 ▸; Nassour *et al.*, 2017 ▸; Dittrich *et al.*, 2004 ▸, 2013 ▸; Volkov *et al.*, 2007 ▸; Dominiak *et al.*, 2007 ▸; Bąk *et al.*, 2011 ▸; Jarzembska & Dominiak, 2012 ▸; Kumar *et al.*, 2019 ▸). Calculation of atomic form factors with this kind of model is already quite fast [*e.g.* about 10 s on one CPU core for small (942 atom) protein crambin (Chodkiewicz *et al.*, 2018 ▸)]. Unfortunately, it is also slightly less accurate than HAR, especially in the case of polar hydrogen atoms because effects of intermolecular interactions on atomic densities are not included in this model as well as the assumptions of transferability of atomic densities and the limitations of the electron density model used in the multipole formalism (Koritsanszky *et al.*, 2011 ▸).

The concept of transferability is also used in the HAR–ELMO method (Malaspina *et al.*, 2019 ▸) which combines libraries of extremely localized molecular orbitals (ELMO) with HAR (Meyer & Genoni, 2018 ▸). Both TAAM and HAR–ELMO require precomputed parameters specific to the atom types present in the molecule of interest. It is also possible to represent part of the system with ELMO and part with higher-level quantum mechanical methods (Macetti & Genoni, 2019 ▸). This approach combined with HAR is known as HAR–QM/ELMO and is used to improve the representation of intermolecular interactions in HAR (Wieduwilt *et al.*, 2021 ▸).

In this work we examine HAR combined with fragmentation on a range of organic and metal–organic systems, in attempts to achieve a considerable reduction of computational time needed for quantum chemical calculations for these systems and preserve the accuracy of the original HAR approach. We also tested the concept of transferability in the case of HAR by transferring atomic electron densities between similar atoms in the structure. This can be thought of as an ‘on the fly’ transferable atom approach.

HAR is a relatively new method and is still undergoing rapid development. A methodology for treating disordered systems was introduced only recently (Kleemiss *et al.*, 2021 ▸). We tested an application of fragmentation for this purpose since it has the potential to isolate the disorder treatment to the fragment of the system in which it is present and can save computational time. We also applied fragmentation to polymeric systems. A typical implementation of HAR includes calculation of the wavefunction for the molecule or cluster of chemical units extracted from a crystal. For polymeric systems, it is not possible to extract such a system without breaking bonds but fragmentation methodology provides tools for handling this.

In HAR, as in quantum chemistry calculations, the user can choose various parameters of the applied method – *e.g.* method of wavefunction calculation, basis set used, method of representing intermolecular interactions and method of atomic density partition (Chodkiewicz *et al.*, 2020 ▸; Wieduwilt *et al.*, 2020 ▸) – yet the optimal choice of HAR settings is still an open question. The way fragmentation of a system is performed adds another dimension to the space of HAR settings. Such settings also influence computational time and the accuracy of the method. In order to optimize the benefits of the fragmentation approach, a balance between accuracy and efficiency needs to be addressed (Khire *et al.*, 2018 ▸). We therefore compared the effects of fragmentation to the effects related to changes in some other parameters of HAR.

## 2. General description of implementation and fragmentation scheme

**Figure 1:**
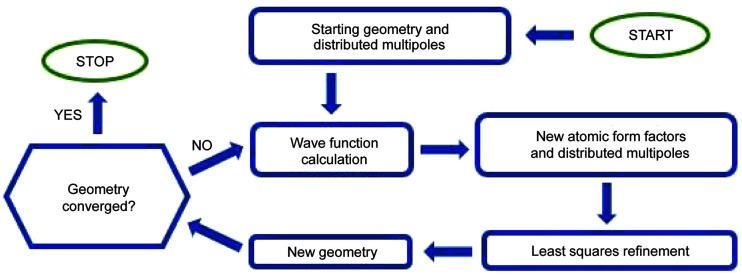
HAR algorithm flowchart.

HAR (Jayatilaka & Dittrich, 2008 ▸) (see Fig. 1 ▸) was implemented in a similar way to the version introduced by Capelli *et al.* (2014 ▸), but there are some differences. Distributed multipoles, which are used for representing a crystal field, are updated on the same foot as atomic form factors (*i.e.* when a new geometry is available, new atomic form factors and distributed multipoles are calculated from the wavefunction calculated for the system in the presence of multipoles from previous iterations). This differs from the original implementation where multipoles are calculated iteratively to convergence before form factor calculations. Initially, distributed multipoles are generated using parameters from a bank of transferable atomic densities (Jha *et al.*, 2020 ▸) defined by the Hansen–Coppens multipole model.

The implementation is based on previous developments (Chodkiewicz *et al.*, 2020 ▸) with some technical changes, *i.e.* electron density partitioning no longer relies on external libraries, and a different numerical integration algorithm and a Becke-type multicenter integration scheme are included for molecular integrals (Becke, 1988 ▸) with unpruned grids (with 770 angular and 99 radial points) with a radial integration grid based on an algorithm by Treutler & Ahlrichs (1995 ▸) and a Lebedev–Laikov grid is employed for spherical integration (Lebedev & Laikov, 1999 ▸).

A locally modified version of *Olex2* (Dolomanov *et al.*, 2009 ▸) was used in the refinements. It incorporated a development version of the *discamb2tsc* program (Kumar *et al.*, 2019 ▸; Chodkiewicz *et al.*, 2018 ▸; Gildea *et al.*, 2011 ▸) based on the DiSCaMB library (Chodkiewicz *et al.*, 2018 ▸) which generates files with atomic form factors in tsc format (Midgley *et al.*, 2019 ▸; Kleemiss *et al.*, 2021 ▸). Such files are then imported into *Olex2* and used in the refinement. The procedure is repeated automatically until convergence of HAR cycles is achieved (Fig. 1 ▸). We assume that HAR iteration converges when the ratio of the maximum parameter shift value to the parameter standard deviation is <0.1. In all refinements, *ORCA* (Neese, 2012 ▸) was used for quantum chemical calculations.

Fragmentation is used in quantum chemistry to speed up calculations by partitioning systems into fragments and performing calculations on the fragments (see Collins & Bettens, 2015 ▸; Raghavachari & Saha, 2015 ▸). Fragmentation for the crystal structure starts with splitting the crystal into separate ions/molecules. This kind of fragmentation is partially supported in the original version of HAR. For structures with multiple chemical units, the original version calculates one wavefunction for a cluster of the chemical units. This is different from our implementation – it is possible to calculate a wavefunction independently for each molecule/ion and the molecules ‘feel’ each other through the electrostatic potential generated by their neighbours (represented by distributed point multipoles). We will refer to such fragmentation as intermolecular fragmentation.

Another level of fragmentation requires breaking of covalent bonds (referred to hereafter as intramolecular fragmentation or simply fragmentation). The broken bonds are then capped with hydrogen atoms. Optionally the fragments can be extended (so they overlap) before being capped (see Fig. 5). The electrostatic interactions are modelled with point multipoles placed on surrounding atoms except for those replaced by the capping hydrogens (*e.g.* Amara & Field, 2003 ▸).

**Figure 2:**
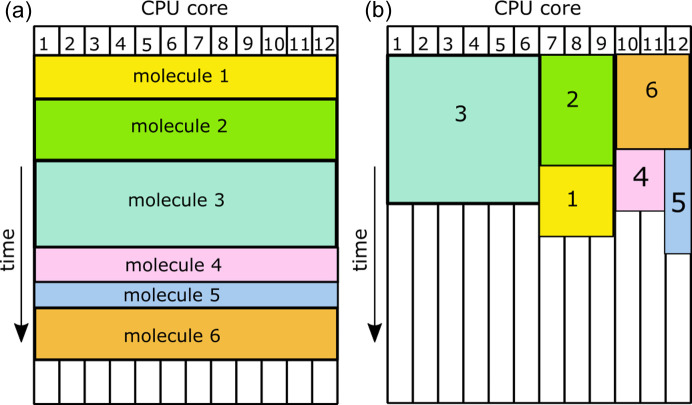
CPU core assignment (*a*) without and (*b*) with load-balancing.

An important factor affecting the calculation efficiency is likely to appear when calculations are performed in parallel. Quantum chemical calculations usually do not scale very well with the number of processor cores (*i.e.* calculations of wavefunction using 10 CPU cores are not usually 10× faster than on 1 core, instead they might only be 2.5× faster in the case of small molecules). A more efficient approach involves simultaneous calculation of multiple wavefunctions [Fig. 2 ▸(*b*)]. We applied a very basic dynamic load-balancing mechanism where the number of CPU cores assigned to particular fragment is defined by the user. The algorithm automatically starts wavefunction calculations for the next fragment when resources are available and it chooses the fragment with the largest number of CPU cores assigned. Development of more automatic and efficient algorithms is planned in the future.

## 3. Quantification of structural differences introduced by fragmentation

Methods that allow faster HAR calculations may lead to inaccuracies due to approximations. In order to assess the accuracy of the method, we monitored the change of *X*—H bond lengths. For some systems we compared the results of HAR refinements with neutron measurements; however, owing to limited availability of this kind of data, only results of regular HAR and HAR with fragmentation are compared. We used multiple datasets available in the literature, the associated references are given in Appendix *A* [App appa] and the compounds are identified with Cambridge Structural Database (CSD, Groom *et al.*, 2016 ▸) refcodes throughout the text (with the exception of popular compounds). The most widely used statistic is an average of the absolute value of the difference in bond length, 〈|Δ*R*|〉. We also used the weighted root mean square difference values for bond lengths, defined as

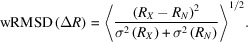

**Figure 3:**
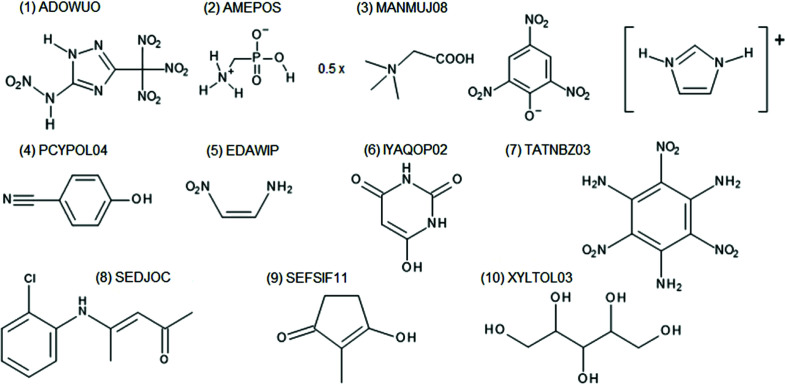
Structures used for testing the influence of the basis set on the *X*—H bond lengths.

**Figure 4:**
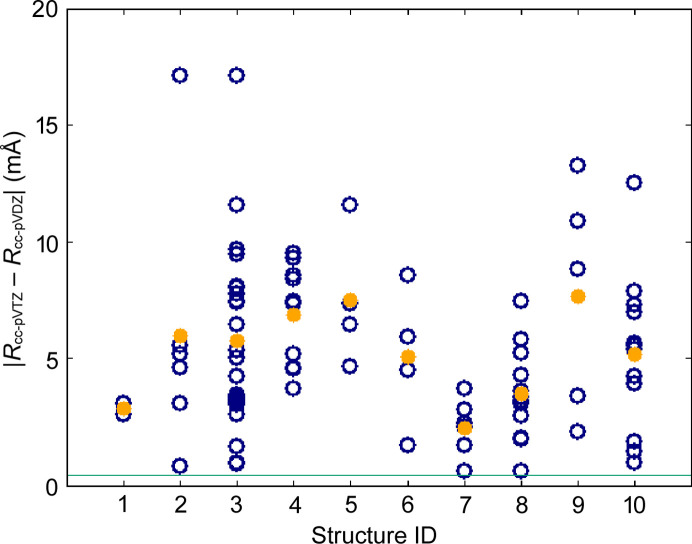
Absolute difference in *X*—H bond lengths for HAR with two different basis sets: cc-pVDZ and cc-pVTZ. Each bond is represented by a dark blue circle. Structure IDs (*x* axis) refer to the labelling in Fig. 3 ▸. Orange circles correspond to the average for a given system.

In order to estimate the relative strength of the effect of fragmentation on the *X*—H bond length it can be compared with the differences introduced by other HAR settings, such as choice of basis set, quantum chemical method and representation of intermolecular interactions. As an illustrative example we compared the results obtained with two basis sets: a smaller cc-pVDZ and a larger cc-pVTZ for ten structures (Fig. 4). The differences in bond lengths are up to 18 mÅ and their averages range from 2.8 to 7.6 mÅ (see Figs. 3 ▸ and 4 ▸). For the different quantum chemical methods the largest differences were observed between Hartree–Fock and density functional theory with BLYP functional, 0.18 mÅ on average for bonds to polar hydrogen atoms (Capelli *et al.*, 2014 ▸; Wieduwilt *et al.*, 2020 ▸; Chodkiewicz *et al.*, 2020 ▸). Lacking representation of intermolecular interactions can also lead to relatively large differences (10–25 mÅ) in bond length on average for polar bonds in xylitol (17.5 mÅ), oxalic acid dihydrate (19 mÅ) and (CSD refcode) MANMUJ08 17 mÅ [Fig. 3 ▸(3)]. In the case of non-polar bonds (C—H) these differences were much smaller (5 mÅ for xylitol and 4 mÅ for MANMUJ08).

All refinements used in this work were performed with the use of density functional theory with B3LYP functional, cc-pVDZ basis set, and a cluster of point charges and dipoles representing the electrostatic field surrounding the molecule of interest lying within an 8 Å range, unless otherwise specified. This basis set is an example of a double-zeta basis set with polarization. This could be a popular choice for HAR calculations as it already provides fairly accurate results in terms of structural parameters derived from HAR. Even when use of a larger basis set is planned, calculations with a smaller one are still useful as a starting point.

## 4. Fragmentation and accuracy

Although the benefits of fragmentation for very large molecules (*e.g.* proteins) are obvious, they might be much less striking for medium-sized molecules. For fragHAR the maximum speed-up for the investigated systems (up to 71 atoms) was ∼2 and even less for the parallel version.

**Figure 5:**
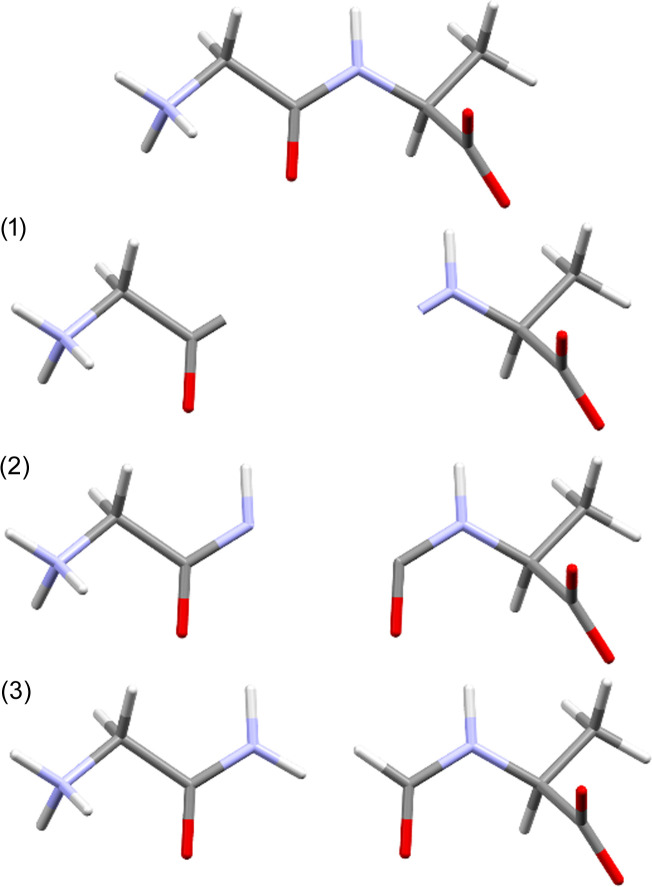
Fragmentation steps: (1) divide a system into separate subunits, (2) extend the subunits so they overlap and (3) add capping hydrogen atoms.

There is a trade-off between speed-up and accuracy in fragmentation. The algorithm for fragmentation should be chosen such that it is more accurate than the TAAM. Otherwise it would make more sense to perform a TAAM refinement as calculations of form factors are faster in this case. TAAM accuracy depends on how accurately the definition of atom types describes the molecular environment of a given atom. Atom-type specification usually contains information on the first neighbours of the atom in terms of chemical element, number of bonded atoms and involvement in an aromatic ring(s). Therefore, such information should be preserved in a fragmentation scheme. One can imagine fragmentation as a three-step process (see Fig. 5 ▸): (1) divide a system into separate subunits, (2) extend the subunits so they overlap and all atoms of the original subunits have their neighbours (at least the first ones) preserved, (3) add capping hydrogen atoms.

We examined what would happen if we used the simplest approach: the above algorithm without extending the initially formed fragments. As a result, the atoms involved in the bond broken when the system was divided do not preserve their neighbours, because the bonding partners are replaced by hydrogen atoms.

**Figure 6:**
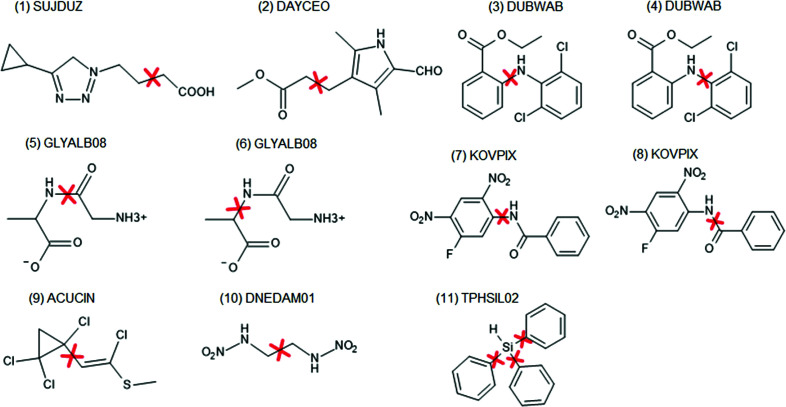
Molecules used for testing fragmentation. The red crosses indicate the bonds where the molecules were split into fragments.

**Figure 7:**
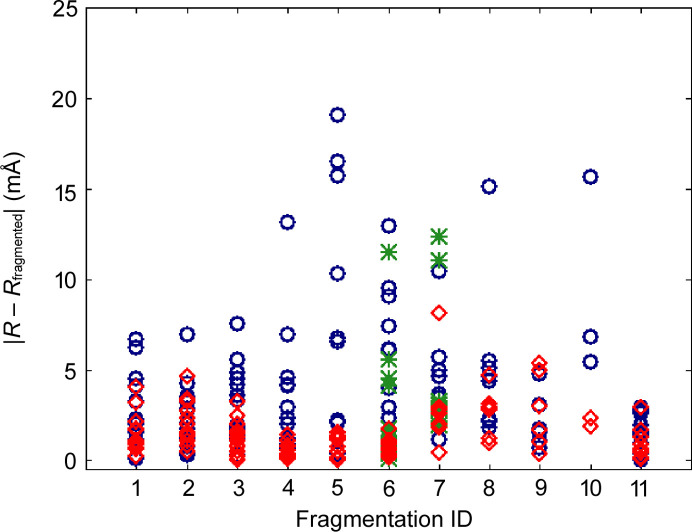
Absolute difference in *X*—H bond lengths for HAR with and without fragmentation. Each bond is represented with (1) a dark blue circle in the case of the more coarse fragmentation scheme and (2) a red diamond for the fragmentation scheme with overlapping fragments that avoids breaking amide bonds. The green stars correspond to *X*—H bonds in the scheme with overlapping fragments which lead to amide bond breaking. Structure ID (*x* axis) refers to the labelling scheme in Fig. 6 ▸.

This approach was tested on 11 cases (Fig. 6 ▸). All of the test molecules were split into two fragments except for the system marked (11) TPHSIL02 which was split into four fragments (corresponding to an SiH_4_ and three benzene molecules). The absolute change in bond length caused by fragmentation is shown in Fig. 7 ▸ (dark blue circles). The difference is quite similar to that caused by switching from the smaller basis set (cc-pVDZ) to the larger one (cc-pVTZ). Lack of representation of the strong hydrogen bonds can easily lead to differences of a similar order.

In order to at least partially mitigate the error introduced by fragmentation, we also tested an approach with overlapping fragments by extending the initial fragments through inclusion of their first-neighbour atoms.

**Figure 8:**
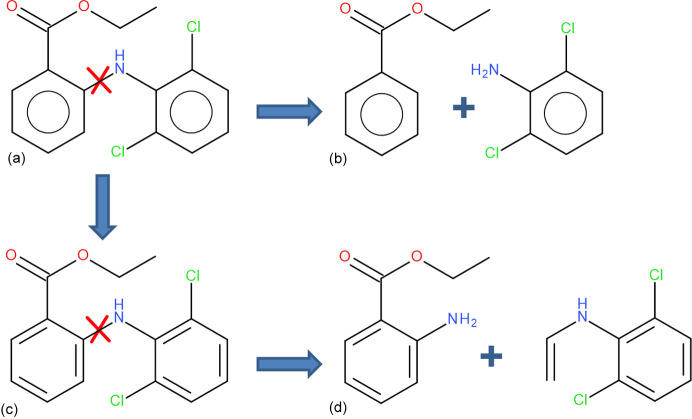
Fragmentation scheme: (*a*) the starting system bond to be broken is marked, (*b*) the system after coarse fragmentation obtained by breaking the bond and adding a capping hydrogen, (*c*) the system with aromatic rings represented as single and double bonds (kekulization was performed), (*d*) the system after fragmentation with the initial fragments extended by at least one neighbour, the aromatic ring was cut along the assigned single bonds in the kekulization procedure.

This involves breaking bonds between the first and second neighbour which is not always possible, *i.e.* when the two are connected by a double bond. In such a case the second neighbour is included in the fragment (or further neighbours if necessary). In the case of aromatic systems such a procedure could lead to quite large fragments, especially when multiple aromatic rings are fused. In order to avoid this, a kekulization procedure was applied in which aromatic bonds [see Fig. 8 ▸(*a*)] were represented as a combination of single and double bonds [see Fig. 8 ▸(*c*)] in such a way that the valences of the involved atoms were satisfied. Then the aromatic bonds assigned as single bonds can be broken [see Fig. 8 ▸(*d*)]. Extending fragments using first-neighbour atoms led to a very significant reduction in the bond length differences with respect to regular HAR (see Fig. 7 ▸, data represented with red diamonds). This happened in all cases except for GLYALB08 and KOVPIX where the extension of the fragments did not lead to better results because (Fig. 7 ▸, data represented with green stars) it involved breaking an amide bond, which is formally a single bond but has partial double-bond character. Inclusion of further neighbours (or simply not breaking the amide bond) led to much better results (red diamonds on the plot). The maximum discrepancy dropped from 19 mÅ for crude fragmentation to 8 mÅ for the overlapping fragments approach (and for all but one *X*—H bond it is no more than 5 mÅ). This difference is already relatively low and indicates that inclusion of the first neighbour extension might be sufficient.

In fact one can expect that, in some situations, even far neighbours can contribute to the fragmentation error, especially if the effect can be transmitted through conjugated bonds. This effect was not strongly present in any of the investigated structures, for example KOVPIX, ACUCIN and DUBVAB (see Fig. 6 ▸), we also checked ADOWUO [see Fig. 3 ▸(1), max difference 2 mÅ], MOSCPO (NO_2_–Ph–COOH, fragmented into NO_2_–Ph and Ph–COOH, 5 mÅ change in O–H bond) and PCYPOL04 [CN–Ph–OH, Fig. 3 ▸(4) fragmented into CN–Ph and Ph–OH]. In one of the two independent CN–Ph–OH molecules the effect was 1 mÅ but for the second it was 8 mÅ. The influence of the substituent effect through an aromatic ring appears to be modest.

### 4.1. Systematic fragmentation method

**Figure 9:**
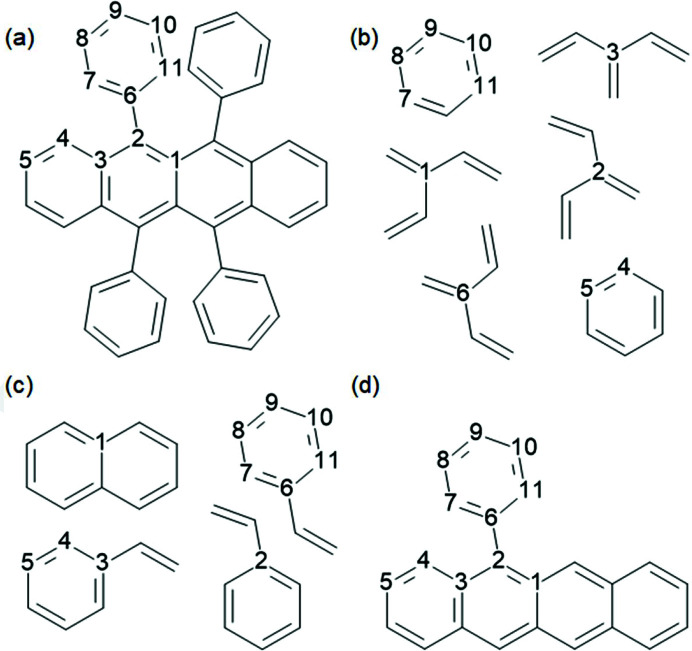
Fragmentation of rubrene according to models (1)–(3) discussed in the text: (*a*) atom labels (when an atomic density for a given atom is taken from a given fragment, the atom in the fragment is enumerated), (*b*) fragmentation according to scheme (1), (*c*) scheme (2) and (*d*) scheme (3).

A more complicated aromatic system – rubrene (Fig. 9 ▸, CSD refcode QQQCIG17) – was chosen for an additional examination of the kekulization based approach for aromatic rings. A general systematic approach for fragmentation was adopted: (1) for each non-hydrogen atom in the asymmetric unit we create a fragment containing the atom and its first-neighbour hydrogen atoms, also adding other atoms according to some additional rules; (2) if the first step involved breaking covalent bonds, hydrogen atoms are attached to the dangling bonds; (3) redundant fragments are removed (multiple copies of the same fragment and fragments that are sub-fragments of larger fragments). Three models of fragmentation were tested, differing in the specification of the additional rule for preserving neighbour atoms used in step (1):

Model (1) The first neighbour is preserved, and if the atom is connected to hydrogen and it belongs to an aromatic ring then all of the atoms forming the ring are also included.

Model (2) The first neighbour is preserved, and if the atom belongs to an aromatic ring then all of the atoms forming the ring are also included.

Model (3) At least one neighbour is preserved and no aromatic/multiple bonds are broken (kekulization is not applied).

The resulting fragmentations are shown in Fig. 9 ▸. In the case of the three fragmentation models [referred to hereafter as model (1), model (2) and model (3)] we also adopted a rule that only C—C and C—N bonds could be broken. So when the first neighbour should be preserved and is connected to an oxygen atom, the oxygen atom is also included in the fragment. An example fragmentation list for the model system (DUBWAB) is shown in Appendix *B* [App appb].

There was only a small difference in the structural parameters between the regular HAR results and for all models of HAR with fragmentation. The maximum difference in C—H bond length was 4.3 mÅ and on average 1.9, 1.6 and 0.8 mÅ for models (1)–(3), respectively. When compared with neutron results for the full molecules, HAR gave only slightly better values for the C—H bond lengths than models with fragmentation [on average 9.5 mÅ with no fragmentation versus 10.7, 11.1 and 10.3 mÅ with fragmentation models (1)–(3)]. The version with fragmentation was 15.4× faster than regular HAR when model (1) was used (∼5 min versus 19 s), 9.7 for model (2) and 4.2 for model (3) (results for parallel execution on 12 CPU cores).

## 5. Fragmentation: examples of application

HAR with fragmentation can be applied to a wide range of systems as we will illustrate with a set of examples. We compared wavefunction calculation times for the approaches with and without fragmentation. We did not measure the time of the whole refinement since it strongly depends on the starting point (initial geometry) and convergence criteria (however, a discussion about the total time of refinement is provided in Section 5.7). The atomic form factor calculation times are also not taken into account, since they strongly depend on the integration grid used. The grid choice itself is not yet optimized in HAR. Wavefunction calculation times are measured only for the first iteration of HAR which is more time consuming than in the next steps. Parallel calculations were performed using 12 cores at a time (out of 16 available cores of the processor, AMD Ryzen 9 3950X).

### 5.1. Fragmentation without breaking covalent bonds

**Figure 10:**
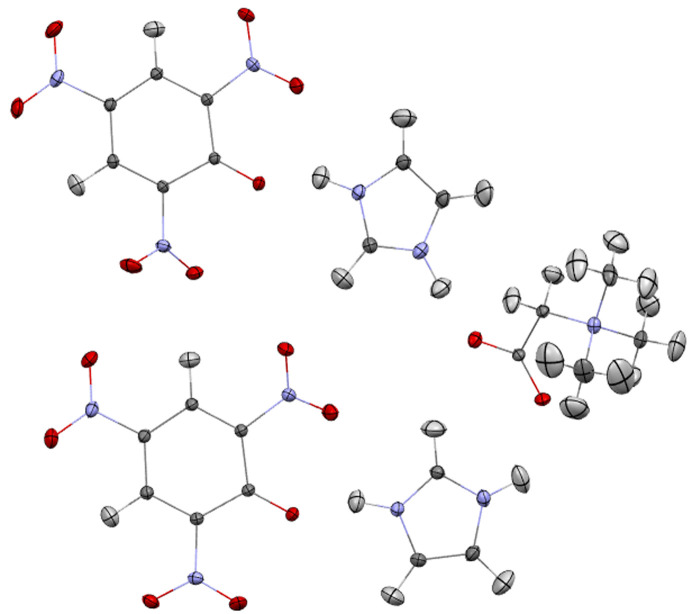
System used for testing fragmentation without breaking covalent bonds (MANMUJ08).

Fragmentation without bond breaking (intermolecular fragmentations) is quite obvious and straightforward but we are not aware of any implementation of this approach which would allow for the handling of intermolecular interactions, which may be important for systems with hydrogen bonds. We applied this type of fragmentation to a compound with five chemical moieties in an asymmetric unit, [CSD code MANMUJ08, see Fig. 3 ▸(3) and Fig. 10 ▸].

Calculation of the wavefunction for a cluster of five chemical units takes 3 min and 50 s and calculations for all wavefunctions independently takes 43 s which is a 5.3-fold acceleration. Representation of intermolecular interactions is less accurate with fragmentation. The discrepancies between neutron and HAR refinements were quite similar. In both cases the average difference in *X*—H bond length was *ca* 16 mÅ. In the case of the N—H bonds which are strongly involved in the intermolecular interactions, the average differences were also very similar, *ca* 14.4 mÅ with fragmentation and *ca* 13.7 mÅ without. Fugel *et al.* (2018 ▸) performed HAR for the structure without a cluster of multipoles, which may be a good approximation since the part of the system treated at a quantum mechanical level does not interact strongly with the surrounding chemical units. The situation is different in the case of intramolecular fragmentation. The lack of distributed multipoles to represent the interactions leads to significantly inferior results. The discrepancy between HAR and neutron values for N—H bonds increased to *ca* 30 mÅ on average (compared with 14 mÅ when the multipoles were included).

### 5.2. Extending fragHAR with inclusion of point multipoles

The original implementation of fragHAR – a fragmentation based HAR approach for proteins and polypeptides – does not use point multipoles, instead electrostatic interactions are modelled using dimers of fragments. We tested the fragHAR approach extended with point multipole representation of electrostatic interactions. In addition, fragmentation model (2) (described in Section 4.1) was examined. In testing cases, we used the tripeptide Ala–His–Ala (AHA; with 2-propanol and water as solvent, CSD refcode DUCMAQ) and the hexapeptide cyclo-(d,l-Pro)2-(l-Ala)4 monohydrate (Pro_2_Ala_4_, CSD refcode CAMVES), both of which were also tested in the original fragHAR paper (Bergmann *et al.*, 2020 ▸).

**Figure 11:**
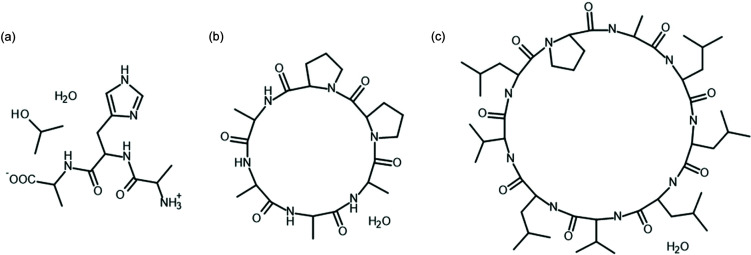
(*a*) Ala–His–Ala tripeptide with 2-propanol and water as the solvents, (*b*) cyclo-(d,l-Pro)2-(l-Ala)4 peptide monohydrate and (*c*) cyclo-Ala–Leu2–VAL–LEU–VAL–LEU–PRO nonapeptide hydrate (LETHIE).

A comparison of bond lengths for refinement with or without fragmentation and with fragmentation without the use of point multipoles is shown in Table 1 ▸. It appears that the effect of fragmentation has a modest impact on the bond length values but the effect of neglecting the intermolecular interaction was relatively large, especially in the case of polar bonds for which the difference resulting from fragmentation alone is on average below 5 mÅ for polar bonds and roughly 3–4× larger when intermolecular interactions are also neglected (no point multipoles are used). Model (2) produces similar results to fragHAR but is based on a coarser fragmentation scheme. These results suggest that a potential source of inaccuracies in TAAM models is more related to neglecting intermolecular interactions than to skipping further covalently bonded neighbours in atom-type definition. The original fragHAR does not involve point multipoles to represent the intermolecular interactions, fragment dimers are used instead. The results show that the application of point multipoles already provides an adequate description of the interactions at a lower computational cost (since there is no need to use dimers). The only slightly worse results obtained with the more coarse-grained model indicate that the model could be a computationally more efficient alternative. The speed-ups are 1.6 and 2.4 in the cases of Pro_2_Ala_4_ and AHA, respectively, when fragHAR is employed, and 2.9 and 3.4 when model (2) is used. This acceleration quickly rises with the size of the system and for the nonapeptide (CSD refcode LETHIE) shown in Fig. 11 ▸ it is 18.6 [for model (2)].

### 5.3. Fragmentation as a tool for handling disorder

**Figure 12:**
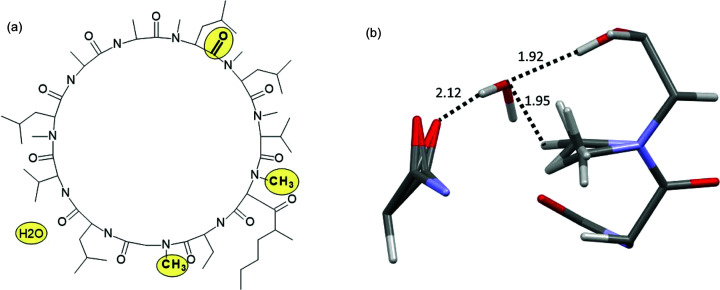
(*a*) Cyclo­sporine A hydrate: disordered sites are marked in yellow; (*b*) structure and interatomic distances between partially occupied atoms (in Å).

While a method for handling disorder in HAR was already proposed by Kleemiss *et al.* (2021 ▸), it relies on the calculation of multiple configurations of the whole disordered system which might lead to quite lengthy calculations, especially for systems with multiple disordered ‘sites’ such as proteins. When a system has two disordered sites with two alternative configurations at each site then there are four combinations of possible configurations – AA, AB, BA and BB – where A and B are variants of configuration. The number of possible combinations grows quickly with the number of disordered sites. Alternatively, if there is no obvious correlation between configurations of the disordered sites, a wavefunction for only two configurations can be calculated (*i.e.* AA and BB). For fragmentation the need for multiple calculations of the wavefunction appears only for the disordered fragments. This approach was tested on the structure of cyclo­sporine A hydrate (LOSLEL), an 11-amino acid cyclic peptide immunosuppressant (C_62_H_111_N_11_O_12_, 0.75H_2_O). There are four disorder sites: three isolated sites within the peptide [see Fig. 12 ▸(*a*)] and a water molecule with partial occupancy. This translates to 2^4^ = 16 possible arrangements of the molecules in the asymmetric unit. A single wavefunction calculation for the polypeptide takes 44 min, amounting to 12 h for all 16 combinations. We have used an alternative approach for the whole-molecule calculations described above: one with all the major components of disorder on each site and one with minor components only takes 85 min to calculate. With model (2) the fragmentation scheme results in 37 fragments, although the wavefunction calculations take only 1 min and 33 s. The difference in *X*—H bond lengths between regular HAR and the version with fragmentation is 4.7 mÅ on average (4.7 mÅ for C—H, 4.5 mÅ for N—H and 3.6 mÅ for O—H).

**Figure 13:**
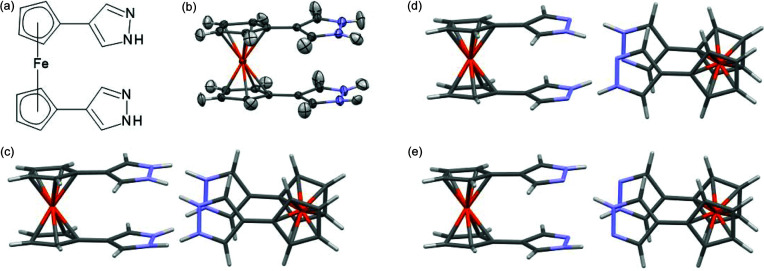
1,10-Bis­(pyrazol-4-yl)ferrocene (BECFAT): (*a*) structural formula, (*b*) average structure with two conformations superimposed (HAR refinement), (*c*) neighboring molecules (average structure), (*d*) one of the conformations expected in the real structure, (*e*) the second, alternative conformation.

Configurations at different disorder sites can also be correlated. For example, in cyclo­sporine A, the occupancy of the H_2_O site and the conformation of the disordered carbonyl group might be correlated since the O⋯H distance in C=O⋯H—O—H is 2.12 Å and the distance between H_2_O and the disordered CH_3_ group is 1.95 Å. These possible correlations between disordered sites have not been explored further for this structure, instead we make no assumptions about such correlations in order to simplify the example. A mean field approximation was used – point multipoles for the given conformation of the disordered site were multiplied by the occupancy factor for this conformation. This approximation should not be used when a correlation between two neighbouring disordered sites is strong and easy to predict. This occurs for another tested structure: a ferrocene derivative (BECFAT, Fig. 13 ▸). The nitro­gen-bonded hydrogen atoms in the structure have 0.5 occupancy. The conformation of one molecule dictates the conformation of its neighbour leading to two possible dimer conformations [see Figs. 13 ▸(*d*) and 13(*e*)]. The wavefunction was calculated for one molecule in each of the two dimers. The influence of the other molecule was represented with point multipoles but without any multiplication of the multipoles by occupancy factors as an assumption was made that atoms of certain conformations were present at 100% probability while others were present at 0% probability so could not serve as centres for point multipoles. However, for neighbouring molecules for which no assumptions are made about the correlation of configurations, the mean field approximation should still be used. Not using the surrounding point multipoles resulted in shorter N—H bonds on average by ∼26 mÅ, and using them but neglecting correlations between the disorder sites led to a similar effect (N—H bonds were shorter on average by 25 mÅ).

### 5.4. Polymeric system

**Figure 14:**
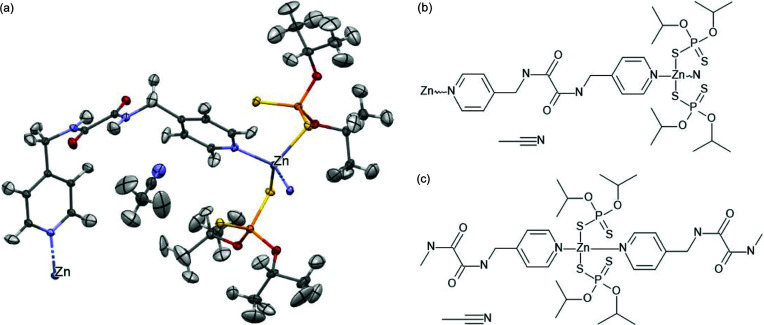
Polymeric system (XUGSEA): (*a*) structure from HAR, (*b*) structural formula, (*c*) fragments used in HAR.

Fragmentation is a natural approach for treating polymeric systems such as metal–organic frameworks (MOFs). An approach similar to fragmentation was used in HAR for CaF_2_ (Kleemiss *et al.*, 2021 ▸) and it involved breaking Ca—F bonds with no further treatment except adjustment of the total spin of the system. Here fragmentation was applied to a metal–organic compound [XUGSEA, Fig. 14 ▸(*b*)] containing hydrogen atoms and typical covalent bonds. The applied procedure was a regular fragmentation [Fig. 14 ▸(*c*)] involving capping broken covalent bonds with hydrogen and performing HAR [Fig. 14 ▸(*a*)].

An alternative approach was also tested. The calculations were performed for the same fragment but without capping broken covalent bonds with hydrogen. It is still possible to perform closed-shell DFT calculations for such systems, but they did not converge within default ORCA limits: 125 SCF iterations which converged after 11 iterations in the case of the capped system. We also tested open-shell approaches, setting the spin multiplicity to 3. In the case of both unrestricted Kohn–Sham (UKS) and restricted open-shell Kohn–Sham (ROKS) the calculations took more time than in the case of the capped system [restricted Kohn–Sham (RKS) calculations]. The execution times were UKS 19 min and 45 s, ROKS 28 min and 1 s, and RKS 15 min and 1 s. HAR with the open-shell DFT approach was performed using UKS. The resulting *X*—H bond lengths were very similar to those for the capped system as the differences did not exceed 0.6 mÅ.

### 5.5. Influence of fragmentation on computational time

Even with quite small fragments it is possible to perform HAR which results in structural parameters that are very similar to those obtained from regular HAR. This significantly speeds up calculations in the case of larger systems as the time needed for the wavefunction calculations for many small fragments should be less than the time needed for the calculation of the whole system simultaneously. This is a consequence of unfavourable scaling of quantum chemistry methods used in HAR. However, there might also be an opposite effect when the calculations are run in parallel.

In the ideal situation calculations performed with *N* CPU cores are *N*× faster. In practice, the acceleration is smaller and efficient use of many CPU cores is more difficult to achieve for small systems. This problem arises in the case of fragmentation as it leads to relatively small systems. This was observed in the fragHAR calculations (Bergmann *et al.*, 2020 ▸). It is especially visible when the simplest naive approach is used whereby calculations are performed for one fragment at a time using all CPU cores. A remedy for this situation is to run multiple calculations of the wavefunction at the same time – each calculation uses only a fraction of the total number of CPU cores (load-balanced approach, shown in Table 2 ▸). Calculations with only 1 CPU core can be treated as a reference since they allow for the largest acceleration because the problem of efficient use of multiple CPU cores does not appear here. For multiple CPU cores the acceleration can only be smaller. Typically, the smaller the fragments are, the larger the drop in acceleration. This is illustrated with calculations for rubrene where models of varying fragment size were used. The smallest fragments gave 20-fold acceleration in the case of 1 core but only 6.3 when the naïve approach was employed with 12 cores. This improved to 15.3-fold acceleration when a load-balanced approach was used. For the case of larger fragments the difference in acceleration is smaller: 6.3 and 9.7 for the naïve and load-balanced approaches, respectively.

Similarly in the case of Pro_2_Ala_4_ and AHA we applied two fragmentation approaches – fragHAR and model (2) (resulting in smaller fragments than fragHAR). The performance of the calculations for model (2) was more sensitive to the choice of CPU core assignment algorithm; in the case of AHA only using a load-balanced approach allowed for better increased acceleration compared with fragHAR (3.4 versus 2.4). In the case of cyclo­sporine A, the acceleration increased from 14- to 55-fold after switching from the naïve to the load-balanced algorithm. Since the structure was divided into a large number (37) of relatively small fragments of similar size, it was in fact quite easy to gain optimal acceleration by assigning one core to each of the fragments for the calculation.

### 5.6. Systems with metal–hydrogen bonds

**Figure 15:**
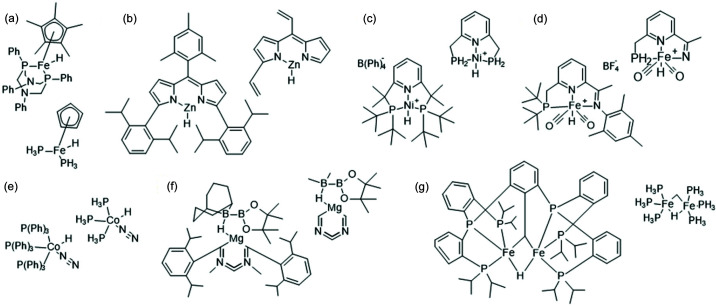
Metal–organic structures (whole system + fragment containing metal atom): (*a*) AHUKIZ, (*b*) LOKPOT, (*c*) MOVPIZ, (*d*) MUHBOI, (*e*) PPHCHN11, (*f*) QEPCOG, (*g*) YULKIC.

Large structure size is quite common for metal–organic systems, where the metal centre is often surrounded by large ligands. The average number of atoms in the largest moiety in the structure of the organic compound crystals is 56, whereas for metal–organic systems it is 129 (for structures published after 2015, crosschecked with the CSD). We chose seven metal–organic systems with metal–hydrogen bonds for tests (see Figs. 15 ▸ and 16 ▸). Fragmentation was performed on the basis of chemical intuition, without the use of any systematic fragmentation (fragmentation schemes are included in the supporting information). The results obtained are presented in Table 3 ▸. For the tested metal–organic systems the acceleration seemed to be smaller (3–7.6) than for the organic systems of similar size.

It appears that the most time-consuming part of HAR calculations with fragmentation is related to fragments that contain metal atoms. This is not surprising since metal compounds are known to be more challenging in terms of SCF convergence, especially open-shell systems. The only open-shell system (YULKIC, *S* = 1) tested was the most expensive computationally. In this case the wavefunction calculation took 3 h and 17 min, and 31 min for the version with fragmentation. The maximum difference for the metal–hydrogen bond lengths was 12.7 mÅ and the maximum wRMSD for this kind of bond was 0.7 (Table 3 ▸). It is not uncommon that the differences between HAR and neutron values for transition metal–hydrogen bond lengths are significantly larger (Woińska *et al.*, 2021 ▸), therefore the effect of fragmentation seems to have a rather minor to modest effect on the accuracy of these values.

### 5.7. Comment on the total time of refinement

The time-consuming steps of HAR performed in this work involve (1) calculation of the wavefunction, (2) calculation of atomic densities at integration grid points, (3) calculation of atomic form factors and saving the file with the form factors, and (4) reading the file with the form factors and least-squares refinement.

We have reported the time related to the calculation of a wavefunction in the first step of HAR. Here we report the total time of refinement and times related to particular steps of refinement for some systems. Note that some of the steps are not yet speed-optimized. As a result, the execution time may change. Calculation of the atomic form factors [step (3)] can potentially be accelerated by reducing the number of points used in numerical integration. Integration grids smaller than that used in this work were already used in HAR. For example, application of the grid used by Capelli *et al.* (2014 ▸) to cyclo­sporine A would lead to about 6.5× fewer grid points compared with the grid used in this work and to 6.5× faster calculations in this step. Moreover, the number of points could probably be reduced further (about 3×) in a similar way to what is done in density functional theory calculations by using so called pruned grids (Gill *et al.*, 1993 ▸; Chien & Gill, 2006 ▸; Dasgupta & Herbert, 2017 ▸). Therefore steps (2) and (3) can probably be performed about 20× faster than in this work. Least-squares refinement [step (4)] depends on the implementation of the algorithm and may vary from program to program; however, more importantly, codes for macromolecular refinement usually use different optimization techniques to those for small molecules and execution times also differ and may scale differently with the size of the system. Moreover each least-squares refinement in this work is performed until (parameter shift)/(standard deviation) drops below 0.001 (default threshold for *Olex2*), which in fact should be adjusted for the analogous parameter for HAR iteration [repetition of steps (1)–(4), see also Fig. 1 ▸]. This could likely speed up step (4).

In the case of HAR with fragmentation, steps (1) and (2) scale roughly linearly with the number of atoms in the asymmetric unit. The time needed for performing step (3) is proportional to (number of atoms) × (number of reflections). The number of reflections which can be theoretically measured is roughly proportional to the volume of the unit cell. Assuming that the number of atoms ( 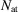 ) is also proportional to the volume of the unit cell, step (3) should scale with 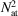 . Full-matrix least-squares refinement [step (4)] involves step scaling as 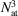 and therefore other methods are usually used in the case of macromolecules (*e.g.* Tronrud, 2004 ▸). Hence for large systems the full-matrix least-squares refinement will be the most time-consuming step and, for other methods, it can be the step related to the calculation of atomic form factors [step (3)].

Timings for steps (1), (3) and (4) are shown in Table 4 ▸. The time for step (2) is not reported here since it is relatively short. Load-balancing in step (1) is not yet built into the refinement procedure and we report the estimated total time of refinement corresponding to the version with the load-balancing incorporated into HAR (details are given in the supporting information). Additional related data [including step (2) timing, real refinement times, timing for wavefunction calculation without load-balancing] are reported in Table S2 of the supporting information. The starting geometries for all refinements were taken from the TAAM refinement performed with the UBDB2018 databank using *Olex2* (Dolomanov *et al.*, 2009 ▸). TAAM form factors were generated with the help of *discamb2tsc* and subsequently imported into *Olex2* in the form of tsc files (Midgley *et al.*, 2019 ▸; Kleemiss *et al.*, 2021 ▸).

If the assumption about the possibility to speed up step (3) about 10–20× is correct the step should not be a computational bottleneck for any of the refinements reported in Table 4 ▸ and the most time-consuming steps are either the wavefunction calculation or least-squares refinement. Yet, because of its scaling properties, step (3) may become the slowest in the case of macromolecules. Step (3) strongly depends on data resolution. In the case of rubrene and Pro_2_Ala_4_, limiting the resolution to 0.8 Å led to a significant reduction in the time needed to perform the step. For the largest system in the table, cyclo­sporine A, the slowest step is clearly the least-squares refinement. For an organometallic system (MUHBOI), the wavefunction calculation is the slowest step. It is clear that in all cases fragmentation significantly reduced the computational time.

**Figure 17:**
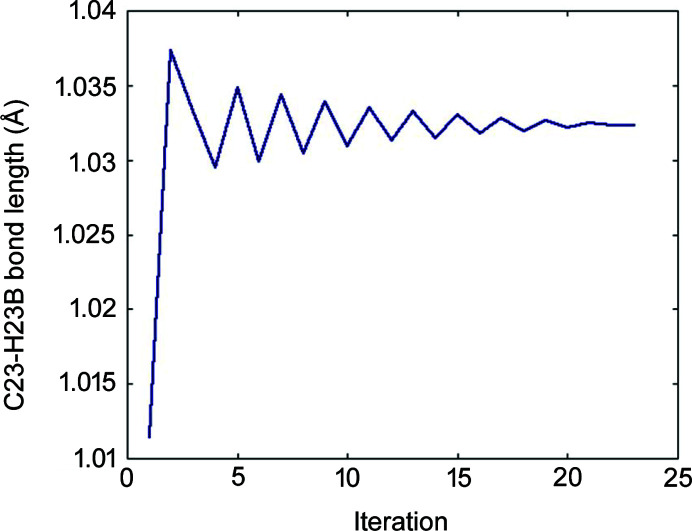
Oscillatory behaviour of the C23—H23B bond length (MUBOI structure) during HAR (for the version with fragmentation).

HAR can involve many iterations. Choice of convergence threshold is a very important factor influencing HAR timing and there is not a well established procedure for choosing it. Refinement of MUHBOI required as many as 22 HAR iterations in the case of HAR with fragmentation and slightly fewer (15×) for HAR without fragmentation. We noticed that some structural parameters (*e.g.* bond length; see Fig. 17 ▸) oscillate during the refinement. Similar behaviour has been observed previously (Chodkiewicz *et al.*, 2020 ▸) and *ad hoc* solutions that depend on choosing an average geometry led to rapid convergence. The oscillatory behaviour might be potentially related to the fact that, during least-squares refinement, some of the terms arising from the change of atomic densities with the change of geometry are usually ignored when calculating derivatives of structure factors with respect to atomic coordinates (Midgley *et al.*, 2021 ▸). The problem of oscillatory behaviour in HAR has not yet been explored.

## 6. Transferability of atomic electron densities

**Figure 18:**
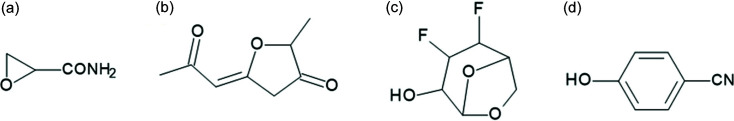
Structures with two molecules per asymmetric unit for which HAR with transferable atoms was applied: (*a*) ARAQUH, (*b*) SIFBAN, (*c*) EGUFIY, (*d*) PCYPOL04.

Further gains in computational acceleration can be obtained with an approach that is halfway between HAR and the use of transferable atomic densities stored in the databank (TAAM approach). In this approach, atomic electron densities obtained for one fragment/moiety are transferred to atoms in another part of the system when the chemical environment of the atom matches. Herein we will refer to the approach as HAR with transferable atoms (HARwTA). Similar to the case of TAAM, a local coordinate system is defined for each atom and transfer of the atomic density involves rotation of the atomic density. The straightforward application of this approach uses HAR for systems with two or more symmetry-independent molecules of the same compound – the wavefunction is calculated for only one of them and the atomic densities are transferred to the other molecule(s). We tested it on the four systems shown in Fig. 18 ▸. The difference in bond lengths between the structures from regular HAR and HARwTA for these compounds was quite small (Table 5 ▸). Statistics were calculated only for the atoms to which the atomic electron densities were transferred (*i.e.* not those for which the atomic electron density is obtained directly from the wavefunction for the moiety containing a given atom). The largest average difference in bond length for this group of molecules was 4.3 mÅ. We also tested the approach for the transfer of atomic densities within a fragmentation scheme in which the electron density is calculated for only some of the fragments and then transferred to chemically similar atoms in other fragments. For SiH(Ph)_3_ the densities were transferred from one phenyl group to two others and for Pro_2_Ala_4_ from one proline fragment to another and from one alanine fragment to the other alanine fragments. For HARwTA combined with fragmentation, results were compared with HAR with fragmentation [option (a) in Table 5 ▸] and without fragmentation [option (b)]. The difference is quite small in the case of SiH(Ph)_3_ – the average discrepancy in C—H bond lengths is only 2.2 mÅ [option (a)] and 1.8 mÅ [option (b)], but for N—H bonds in Pro_2_Ala_4_ it is more significant – 17 mÅ on average when compared with HAR with no fragmentation. For polar hydrogen atoms, we can expect that transferability strongly depends on the hydrogen bonding of the transferred atom. For systems containing two or more symmetrically non-equivalent small molecules of the same compound in the asymmetric unit, the hydrogen bond situation is often very similar for all molecules in the system and this is the case for the four tested structures of this kind. For three systems the intermolecular interactions were shown to be quite important; HAR without representation of these interactions gave visibly different polar bond lengths (see Table S1), but there was no such difference in the refinement with atomic density transfer which meant the effect of the interactions was transferable for these systems. In the case of Pro_2_Ala_4_ the situation was different; the N—H⋯O hydrogen bond in the original fragment is directed toward the carbonyl in the amide group but the atomic density is transferred to the hydrogen atom involved in a hydrogen bond with a water molecule. This, together with the effect of fragmentation and the differences in molecular environment, leads to the considerably larger deviation in bond lengths than for other systems. Overall, combining HAR with atomic density transfer works very well, even for systems with polar hydrogen atoms, as long as the densities are transferred between atoms with a matching molecular environment (including hydrogen bonds).

## 7. Conclusions

We tested an application of a fragmentation method in combination with HAR. Fragmentation facilitated the acceleration of the slowest step in HAR, the wavefunction calculation and also allowed for a natural treatment of polymeric systems. Combining HAR and the TAAM by allowing for the transfer of atomic densities derived from fragment wavefunctions to similar atoms in other fragments meant that the fragmentation-related acceleration was gained at the cost of only a minor difference in structural parameters when compared with standard HAR. Both fragmentation and TAAM are based on the idea of transferability. Fragmentation-based refinements allowed insight into possible ways of improving TAAM. The main points of the work can be summarised as follows:

The slowest step in HAR refinement – the wavefunction calculation – was slightly accelerated for (not very) small molecules without a significant change in *X*—H bond lengths (*e.g.* 2.9× for Pro_2_Ala_4_, 3.4× for Ala–His–Ala solvate); the acceleration increases for larger systems [*e.g.* 18.6× faster for nonapeptide (C_48_H_85_N_9_O_9_·H_2_O), 9.7–15.6× (depending on the approach) for rubrene C_42_H_28_ (also because of its symmetry) and 55× for disordered 11-amino acid-large cyclo­peptide cyclo­sporine A (85 min versus 1 min and 33 s.)]. For the metal–organic systems tested, the acceleration did not increase as much (3–7.6-fold) as it was limited by slow calculations for the fragment containing metal. Higher accelerations can be achieved for symmetric molecules (calculations for only part of the molecule is required) such as rubrene.

Fairly accurate results were obtained when fragments with overlapping atoms were used. In the test of eight small molecules, the maximum difference in bond length compared with regular HAR was only 5 mÅ when such an approach was applied. For comparison, switching from the cc-pVTZ to the cc-pVDZ basis set for other sets of small molecules led to differences in bond lengths of up to 18 mÅ and their averages (for *X*—H bonds for each molecule) ranging from 2.8 to 7.6 mÅ. Even larger differences were caused by neglecting intermolecular interactions. Therefore, introducing representations of intermolecular interactions to the TAAM model seems to be one of the main approaches used to improve the model, whereas providing more specialized atom types in the existing atomic density databanks might be less effective. This also means that, in the case of fragments not involved in strong intermolecular interactions, it could be possible to replace wavefunction calculations by the TAAM approach based on Hirshfeld atomic densities without a drop in accuracy [supported by results for combined HARwTA refinements].

Our implementation allows for an efficient handling of intermolecular interactions between fragments. This was shown to be an important factor influencing the accuracy of refinement and allowed for more efficient fragHAR performance (a fragmentation approach for polypeptides). The approach allows for a very basic but important type of fragmentation – intermolecular fragmentation – involving splitting the calculations of wavefunction for asymmetric units, which include multiple chemical units, into separate calculations for each of the chemical units and ensuring that the effects of interactions between the chemical units are modelled.

Due to the abundance of aromatic rings in organic structures it is important to use an efficient methodology to handle the fragmentation of systems containing them. This can be effectively done using the kekulization method by writing the aromatic part as a combination of single- and double-bonded atoms and breaking the single bonds that appear as a result of this procedure.

In the case of multicore processors, it appears essential for efficiency to perform multiple simultaneous calculations of wavefunctions of fragments. This approach allowed for 3–4-fold acceleration for some systems when 12 CPU cores were used.

Fragmentation is a straightforward approach for performing HAR for polymeric systems. However in the tested system, breaking covalent bonds without capping them with hydrogen atoms resulted in a very similar geometry to the case where hydrogen atoms were used for capping the broken bonds. The version without capping required open-shell calculations which were slightly longer (by 32%).

Combining HAR with TAAM by transferring atomic densities derived from fragments to similar atoms in other fragments allowed for additional acceleration as wavefunction calculations are not needed for all fragments. This approach gave results very similar to standard HAR provided the transfer was performed between chemically similar atoms involved in similar intermolecular interactions.

We performed several tests for one of the proposed fragmentation models [model (2)] and achieved a considerable acceleration for larger systems at the cost of only minor differences in structural parameters. However, the optimal method for fragmentation is not yet established. Therefore, for the next step towards application of fragmentation in HAR, we plan to develop software for automatic fragmentation that would allow for both straightforward application of the procedure and exploration of its variants which would aid the selection of the optimal method of fragmentation for use with HAR.

## Supplementary Material

Supporting information. DOI: 10.1107/S2052252522000690/fc5059sup1.pdf

Zipped cif files. DOI: 10.1107/S2052252522000690/fc5059sup2.zip

CCDC references: 2155274, 2155275, 2155276, 2155277, 2155278, 2155279, 2155280, 2155281, 2155282, 2155283, 2155284, 2155285, 2155286, 2155287, 2155288, 2155289, 2155291, 2155292, 2155293, 2155294, 2155295, 2155296, 2155297, 2155298, 2155299, 2155300, 2155301, 2155302, 2155303, 2155304, 2155305, 2155306, 2155307, 2155308, 2155309, 2155310, 2155311, 2155312, 2155313, 2155314, 2155315, 2155316, 2155317, 2155318, 2155319, 2155320, 2155326, 2155327, 2155328, 2155329, 2155330, 2155331, 2155332, 2155333, 2155334, 2155335, 2155336, 2155337, 2155338, 2155339, 2155340, 2155341, 2155342, 2155343, 2155344, 2155345, 2155346, 2155347, 2155348, 2155349, 2155350, 2155351, 2155352, 2155353, 2155354, 2155355, 2155356, 2155357, 2155358, 2155359, 2155360, 2155361, 2155362, 2155363, 2155364, 2155365, 2155366, 2155367, 2155368, 2155369, 2155370, 2155371, 2155372, 2155373, 2155374, 2155375, 2155376, 2155377, 2155378

## Figures and Tables

**Figure 16:**
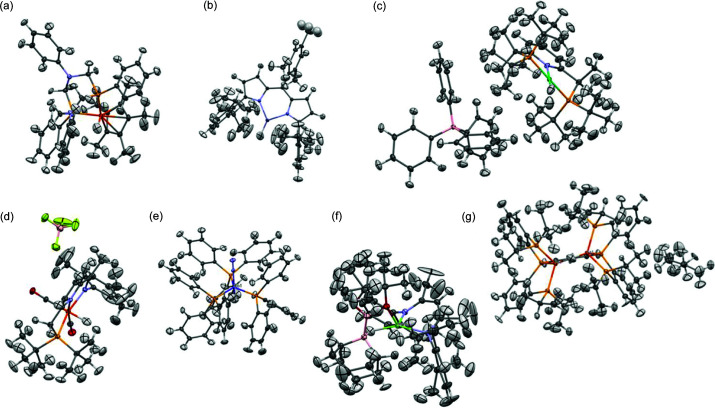
Structures of the metal–organic compounds refined using HAR with fragmentation: (*a*) AHUKIZ, (*b*) LOKPOT, (*c*) MOVPIZ, (*d*) MUHBOI, (*e*) PPHCHN11, (*f*) QEPCOG, (*g*) YULKIC.

**Figure 19:**
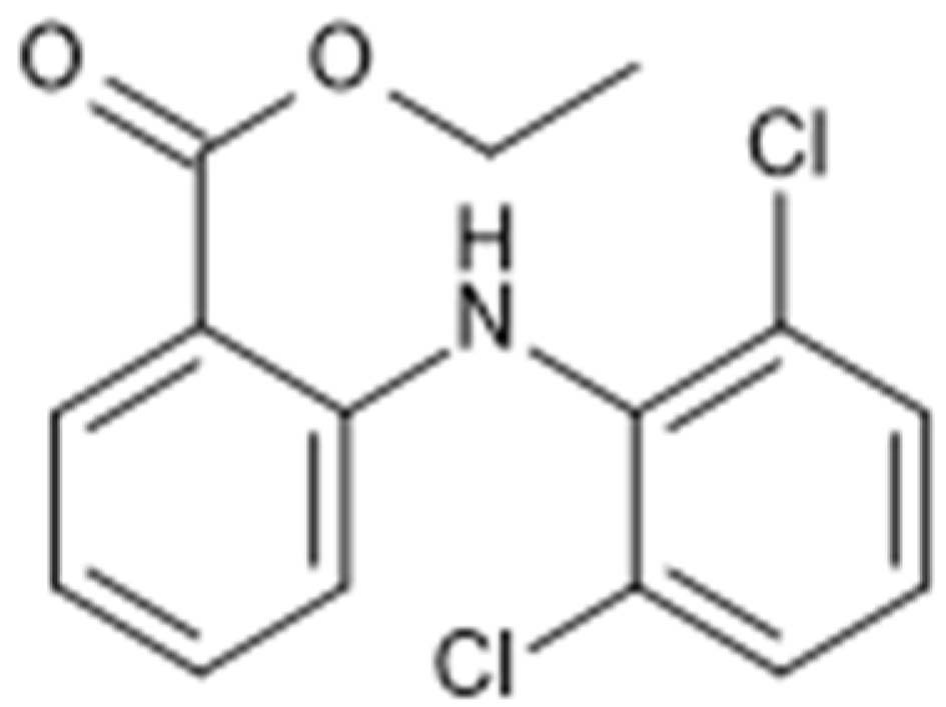
Molecule to be fragmented (DUBWAB).

**Figure 20:**
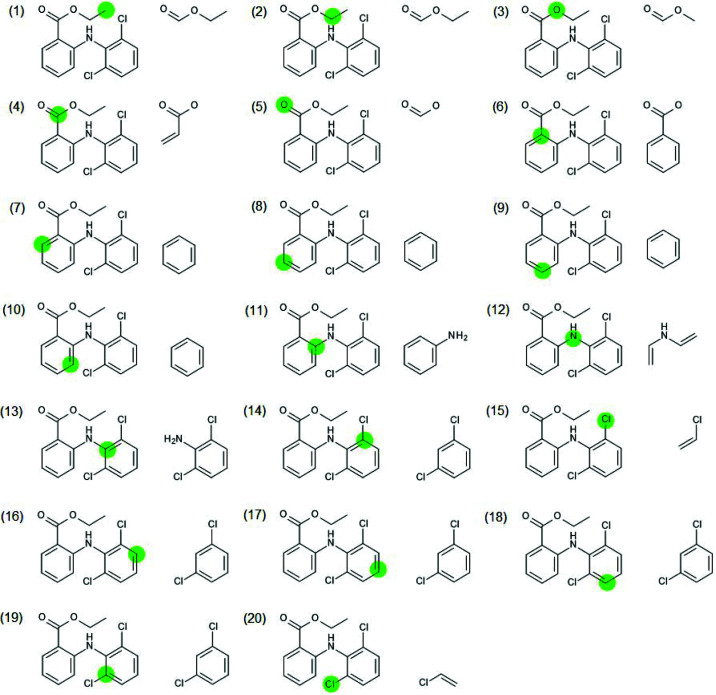
Fragments for each non-hydrogen atom in DUBWAB.

**Figure 21:**
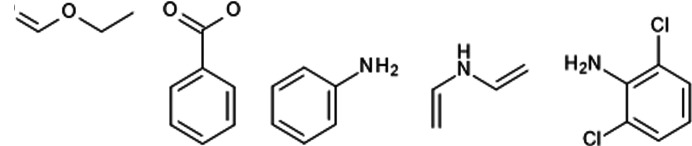
Final set of fragments for DUBWAB.

**Table 1:** Average bond length deviation (〈∣Δ*R*∣〉) and wRMSD (〈∣Δ*R*∣〉) between regular HAR and fragHAR Fragmentation carried out using model (2) and fragHAR without point multipoles [fragHAR(−)]. The data are grouped by bond type.

		〈∣Δ*R*∣〉				wRMSD (〈∣Δ*R*∣〉)			
		*X*—H	C—H	N—H	O—H	*X*—H	C—H	N—H	O—H
Pro_2_Ala_4_	fragHAR	2.1	1.7	4.9	2.8	0.18	0.13	0.39	0.17
	Model (2)	3.4	3.0	5.5	4.7	0.28	0.26	0.38	0.30
	fragHAR(−)	6.1	4.3	14.3	17.0	0.50	0.34	0.99	0.91
AlaHisAla	fragHAR	2.3	2.2	3.4	0.8	0.19	0.19	0.23	0.06
	Model (2)	3.0	2.9	3.8	1.9	0.26	0.27	0.27	0.12
	fragHAR(−)	9.7	6.1	15.9	20.8	0.77	0.47	1.15	1.27

**Table 2:** Wavefunction calculation time and speed-up related to fragmentation for selected systems

System and time (s) without fragmentation (1 core, 12 cores)	Fragmentation model	Speed-up		
		1 core	12 cores naive	12 cores load-balanced
Rubrene (2865, 296)	Model (1)	20.0	6.3	15.3
	Model (2)	13.5	6.6	9.7
SiH(Ph)_3_ (440, 48)	Model (2)	2.3	0.94	1.9
Pro_2_Ala_4_ (1787, 187)	fragHAR	1.8	1.3	1.6
	Model (2)	3.6	1.8	2.9
AHA (849, 93)	fragHAR	2.6	1.3	2.4
	Model (2)	5.3	1.3	3.4
Cyclo­sporine A (NA, 5125)	Model (2)	–	13.8	55

**Table 3:** Results of fragmentation for systems with metal–hydrogen bonds including (1) Calculation time for the non-fragmented system, (2) acceleration, (3) calculation time for the fragment containing metal as a percentage of the total calculation time [% time Frag(Met)], (4) average *X*—H bond length deviation between regular and fragmentation-based HAR and the non-fragmented version of HAR (∣Δ*R*∣), (5) deviation for the metal–hydrogen bond [∣Δ*R*∣ (*M*—H)] and (6) wRMSD for the metal–hydrogen bond (*M*—H).

CSD code	Formula	Time (s)	Speed-up	% time Frag(Met)	∣Δ*R*∣ (mÅ)	∣Δ*R*∣ (*M*—H) (mÅ)	wRMSD *M*—H
AHUKIZ	C_38_H_44_FeN_2_P_2_	1373	3.9	66	6.4	12.7	0.61
LOKPOT	C_42_H_50_N_2_Zn	856	3.4	77	2.1	0.1	0.004
MOVPIZ	C_27_H_52_NNiP_2_^+^, C_24_H_20_B^−^	1057	3.0	63	4.2	3.0	0.41
MUHBOI	C_27_H_38_FeN_2_O_2_P^+^, BF_4_^−^	849	3.3	81	2.6	8.2	0.48
PPHCHN11	C_54_H_46_CoN_2_P_3_	2167	7.6	67	2.9	10.6	0.70
QEPCOG	C_43_H_68_B_2_MgN_2_O_2_	847	5.1	48	4.5	0.3	0.026
YULKIC	C_55_H_76_Fe_2_P_6_, 2C_5_H_12_	11811	6.5	96	3.0	2.6	0.10

**Table 4:** Timings for steps (1)–(4) for a number of the systems used in this work Step (1) timing given for the first iteration of HAR for calculations with load-balancing, time for steps (3) and (4) is an average over HAR iterations, ‘HAR time (estimate)’ corresponds to the estimated time of refinement with the use of load-balancing

		HAR with fragmentation					HAR without fragmentation	
			Time (s)					
System	Resolution (Å)	No. iterations	Step (1) with load-balancing	Step (3)	Step (4)	HAR time (estimate)	No. iterations	HAR time (s)
Cyclo­sporine A	0.55	6	79	369	796	9060	5	34142
Pro_2_Ala_4_	0.38	5	50	141	72	1336	5	1828
	0.80	5	50	16	8	462	4	833
MUHBOI	0.76	22	257	18	7	3724	15	8879
LETHIE	0.82	5	56	30	77	944	4	4847
Rubrene	0.45	6	15	21	11	214	6	2703
	0.80	5	15	4	4	171	6	1939

**Table 5:** Discrepancies between HAR with and without transferability applied The average *X*—H bond length deviation (∣Δ*R*∣; calculated separately for C—H, N—H and O—H) and wRMSD (∣Δ*R*∣); for SiH(Ph)_3_ and Pro_2_Ala_4_ there are values for comparison to HAR with fragmentation [option (a)] and to regular HAR [option (b)].

	∣Δ*R*∣	wRMSD	∣Δ*R*∣ C—H	∣Δ*R*∣ N—H	∣Δ*R*∣ O—H
ARAQUH	2.6	0.14	3.6	1.0	–
SIFBAN	3.9	0.40	4.2	1.0	–
EGUFIY	2.9	0.24	3.0	–	2.3
PCYPOL04	1.5	0.25	1.4	–	1.8
SiH(Ph)_3_ (a)	2.2	0.32	2.2	–	–
SiH(Ph)_3_ (b)	1.8	0.28	1.8	–	–
Pro_2_Ala_4_ (a)	6.3	0.47	6.0	8.6	–
Pro_2_Ala_4_ (b)	7.3	0.58	6.2	17.0	–

## Appendix A References to experimental data used in this work

Sorted alphabetically by CSD refcodes: ACUCIN (Nikonova *et al.*, 2018 ▸), ADOWUO (Hermann *et al.*, 2017 ▸), AHUKIZ (Weber *et al.*, 2015 ▸), AMEPOS (Janicki & Starynowicz, 2010 ▸), ARAQUH (Hemgesberg *et al.*, 2016 ▸), BECFAT (Veronelli *et al.*, 2017 ▸), CAMVES (Dittrich *et al.*, 2002 ▸), DAYCEO (Uppal *et al.*, 2017 ▸), DNEDAM01 (Gruhne *et al.*, 2021 ▸), DUBWAB (Nugrahani *et al.*, 2019 ▸), DUCMAQ (Grabowsky *et al.*, 2009 ▸), EDAWIP (Evans & Gilardi, 2001 ▸), EGUFIY (Malassis *et al.*, 2019 ▸), GLYALB08 (Capelli *et al.*, 2014 ▸), IYAQOP02 (Marshall *et al.*, 2016 ▸), KOVPIX (Thanzeel *et al.*, 2019 ▸), LETHIE (Chen *et al.*, 2018 ▸), LOKPOT (Ballmann *et al.*, 2019 ▸), LOSLEL (Johnas *et al.*, 2009 ▸), MANMUJ08 (Jorgensen *et al.*, 2014 ▸), MOSCOP (Clegg, 2019 ▸), MOVPIZ (Lapointe *et al.*, 2019 ▸), MUHBOI (Butschke *et al.*, 2015 ▸), OXACDH46 (Kamiński *et al.*, 2014 ▸), PCYPOL04 (Mohanraj *et al.*, 2018 ▸), PPHCHN11 (Scheuermann *et al.*, 2015 ▸), QEPCOG (Pécharman *et al.*, 2017 ▸), QQQCIG17 (Jorgensen *et al.* 2014 ▸), SEDJOC (Venter *et al.*, 2012 ▸), SEFSIF11 (Nassour *et al.*, 2014 ▸), SIFBAN (Mills *et al.*, 2018 ▸), SUJDUZ (Díaz-Peralta *et al.*, 2020 ▸), TATNBZ03 (Chua *et al.*, 2017 ▸), TPHSIL02 (Hupf *et al.*, 2019 ▸), XUGSEA (Tan & Tiekink, 2020 ▸), XYLTOL03 (Madsen *et al.*, 2004 ▸), YULKIC (Arnett & Agapie, 2020 ▸)

## Appendix B Example of fragmentation with model (2)

An example of fragmentation for a model system (DUBWAB, Fig. 19 ▸) using model (2) rules is shown. For each of the 20 non-hydrogen atoms a fragment is created (Fig. 20 ▸). Only non-redundant fragments (Fig. 21 ▸) are used in HAR, *i.e.* redundant fragments.
