# Supplementary material for: Fragmentation and transferability in Hirshfeld atom refinement
Source: IUCrJ. 2022 Feb 26;9(Pt 2):298–315. doi: 10.1107/S2052252522000690 (PMC8895009; doi:10.1107/S2052252522000690)
Supplement: Supplementary file 1 [file m-09-00298-sup1.pdf]

IUCrJ

Volume 9 (2022)

Supporting information for article:

Fragmentation and transferability in Hirshfeld atom refinement

Michał Chodkiewicz, Sylwia Pawlędzio, Magdalena Woińska and Krzysztof Woźniak

S1. Fragmentation of metalorganic systems – system + fragments:

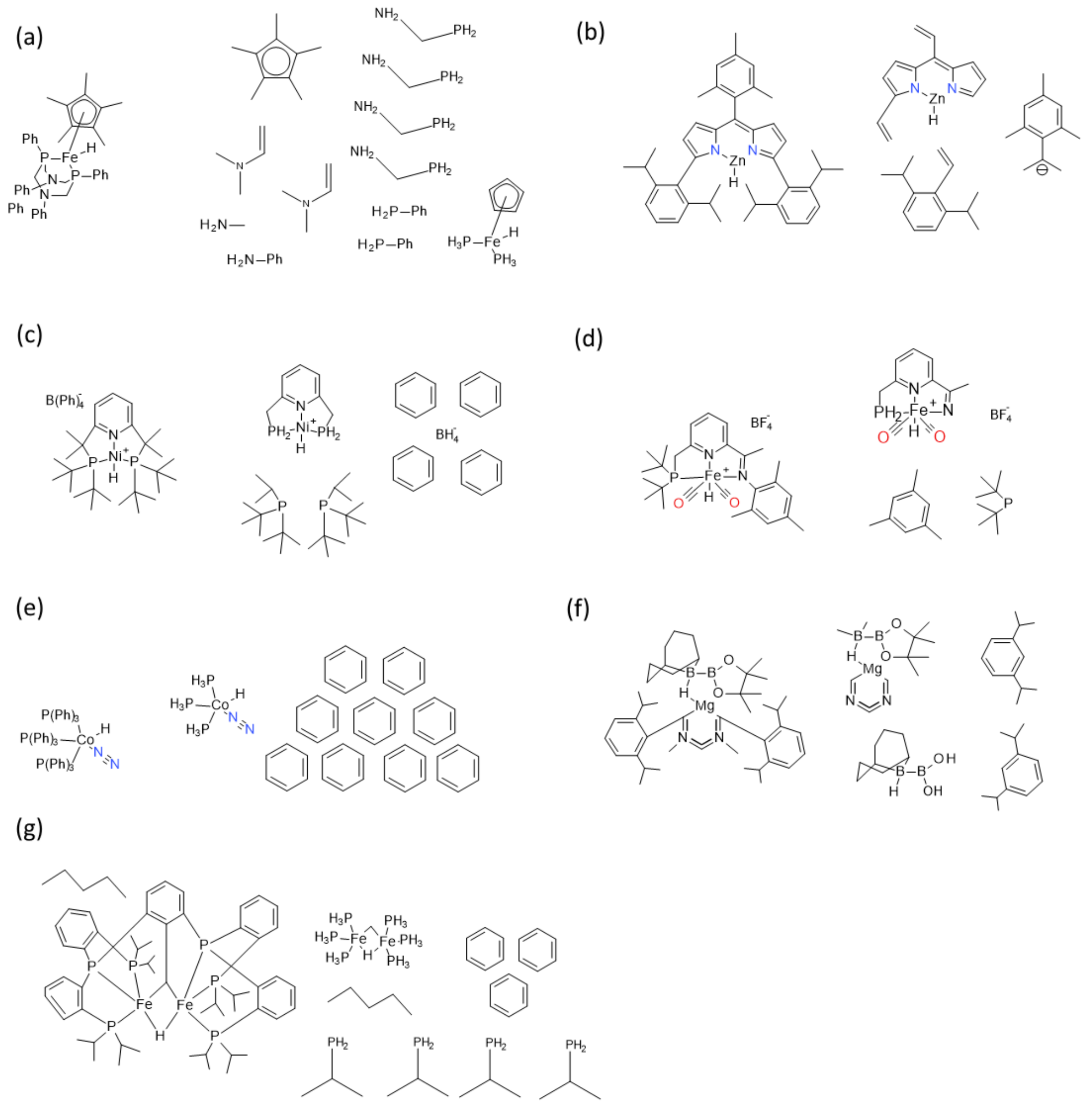

Figure S1 Structures of the metalorganic compounds refined using HAR with fragmentation and the corresponding fragments. (a) AHUKIZ (b) LOKPOT (c) MOVPIZ (d) MUHBOI (e) PPHCHN11 (f) QEPCOG (g) YULKIC

S2. Atomic electron density transfer vs. neglecting intermolecular interactions

The difference in X-H bond lengths between the structures from regular HAR and HAR with transferable atoms (HARwTA) are compared to the differences in regular HAR introduced by neglecting intermolecular interactions (no distributed multipoles used). Statistics were calculated only for the atoms to which the atomic electron densities were transferred (*i.e.* not those for which the atomic electron density is obtained directly from wave function for the moiety containing given atom) and in the case of refinements with no distributed multipoles the same set of atoms as for HARwTA were used in the calculations of statistics.

Table S1 The discrepancies between HAR with and without transferability applied (model: e. d. transfer) and the discrepancies between regular HAR and HAR with no intermolecular interactions represented (model: No multipoles) an average X-H bond length deviation (⟨|ΔR|⟩) (also separately for C-H, N-H and O-H) and weighted root mean square difference wRMSD (⟨|ΔR|⟩), for $SiH(Ph)_3$ and Pro_2Ala_4 there are values for comparison to HAR with fragmentation (an option (a)) and to regular HAR (option (b)).

	Model	⟨|ΔR|⟩	wRMSD	⟨|ΔR|⟩C-H	⟨|ΔR|⟩N-H	⟨|ΔR|⟩O-H
ARAQUH	e. d. transfer	2.6	0.14	3.6	1.0	-
	No multipoles	3.0	0.17	2.0	4.3	-
SIFBAN	e. d. transfer	3.9	0.40	4.2	1.0	-
	No multipoles	2.9	0.35	2.0	9.9	-
EGUFIY	e. d. transfer	2.9	0.24	3.0	-	2.3
	No multipoles	3.4	0.26	2.5	-	9.3
PCYPOL04	e. d. transfer	1.5	0.25	1.4	-	1.8
	No multipoles	5.9	0.94	3.2		16.9

S3. Total times of refinement

Refinements were performed using a 'naïve' approach for calculation of multiple wave functions on multiple CPUs depending on calculation of one wave-function at time. A better version using load balancing approach were used separately from the refinement and similarly as in the case of wave function calculated in the 1-st step of HAR initial guess from previous calculations was not available (i.e. it is reasonable to compare the two times). In order to estimate total time of refinement corresponding to the version with the load balancing being incorporated into HAR (t_{total}^{lb}), a total time of wave function calculation (i.e. in all HAR iterations) using 'naïve' approach (t_{wfn}^{naive}) was

extracted from total time of refinement (t_{total}^{naive}) and total time of wave function calculation using load balanced approach (t_{wfn}^{lb}) were added:

$$t_{total}^{lb} = t_{total}^{naive} - t_{wfn}^{naive} + t_{wfn}^{lb}$$

t_{wfn}^{lb} was also estimated. It was assumed that the proportion between time of wave function calculation with and without load balancing are the same irrespective of HAR iteration. This was used to estimate t_{wfn}^{lb}:

$$t_{wfn}^{lb} = t_{wfn}^{naive} \frac{t_{wfn}^{lb}}{t_{wfn,1}^{naive}}$$

where $t_{wfn,1}^{naive}$ stands for time of wave function calculation without load balancing in the first step of HAR.

Table S2 Data related to refinement timing. Wave function calculation time given for 1-st and 2-nd step of HAR in the case of naïve implementation of parallel calculations, ρ –electron density calculation time, in the case of least squares (LSQ) refinement the time is given for the first and the last HAR iteration. Total refinement time (Real) is given together with estimated time for the refinement with load balancing (Estim.).

System and resolution (Å)		Time (s.)							
		Wave function calc.			ρ	LSQ refinement		Total refinement	
		Naive		Load balancing		first	last	Real	Estim.
		1	2						
Cyclosporine A	0.55	366	348	93	26	1303	391	10530	9060
Pro_2Ala_4	0.38	104	62	64	14	62	99	1471	1336
	0.80	104	62	64	14	11	7	597	462
MUHBOI	0.76	377	209	65	20	7	7	3724	3724
LETHIE	0.82	363	191	65	17	77	60	1682	944
Rubrene	0.45	46	41	19	4	10	9	361	214
	0.80	46	41	19	4	3	3	234	171
